# ADMET evaluation in drug discovery: 15. Accurate prediction of rat oral acute toxicity using relevance vector machine and consensus modeling

**DOI:** 10.1186/s13321-016-0117-7

**Published:** 2016-02-01

**Authors:** Tailong Lei, Youyong Li, Yunlong Song, Dan Li, Huiyong Sun, Tingjun Hou

**Affiliations:** College of Pharmaceutical Sciences, Zhejiang University, Hangzhou, 310058 Zhejiang People’s Republic of China; State Key Lab of CAD&CG, Zhejiang University, Hangzhou, 310058 Zhejiang People’s Republic of China; Institute of Functional Nano and Soft Materials (FUNSOM), Soochow University, Suzhou, 215123 Jiangsu People’s Republic of China; Department of Medicinal Chemistry, School of Pharmacy, Second Military Medical University, Shanghai, 200433 People’s Republic of China

## Abstract

**Background:**

Determination of acute toxicity, expressed as median lethal dose (LD_50_), is one of the most important steps in drug discovery pipeline. Because in vivo assays for oral acute toxicity in mammals are time-consuming and costly, there is thus an urgent need to develop in silico prediction models of oral acute toxicity.

**Results:**

In this study, based on a comprehensive data set containing 7314 diverse chemicals with rat oral LD_50_ values, relevance vector machine (RVM) technique was employed to build the regression models for the prediction of oral acute toxicity in rate, which were compared with those built using other six machine learning approaches, including *k*-nearest-neighbor regression, random forest (RF), support vector machine, local approximate Gaussian process, multilayer perceptron ensemble, and eXtreme gradient boosting. A subset of the original molecular descriptors and structural fingerprints (PubChem or SubFP) was chosen by the Chi squared statistics. The prediction capabilities of individual QSAR models, measured by *q*_ext_^2^ for the test set containing 2376 molecules, ranged from 0.572 to 0.659.

**Conclusion:**

Considering the overall prediction accuracy for the test set, RVM with Laplacian kernel and RF were recommended to build in silico models with better predictivity for rat oral acute toxicity. By combining the predictions from individual models, four consensus models were developed, yielding better prediction capabilities for the test set (*q*_ext_^2^ = 0.669–0.689). Finally, some essential descriptors and substructures relevant to oral acute toxicity were identified and analyzed, and they may be served as property or substructure alerts to avoid toxicity. We believe that the best consensus model with high prediction accuracy can be used as a reliable virtual screening tool to filter out compounds with high rat oral acute toxicity.

**Graphical abstract:**
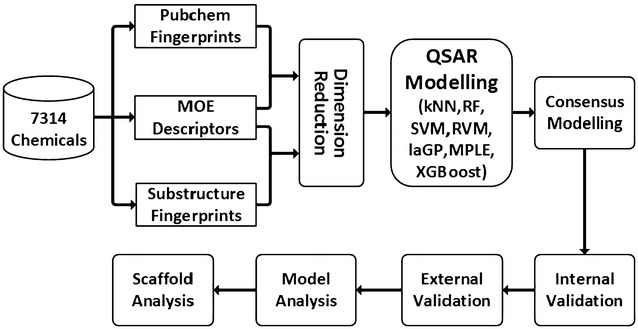
Workflow of combinatorial QSAR modelling to predict rat oral acute toxicity

## Background

Determination of acute toxicity in mammals (e.g. rats or mice) is one of the most important tasks for the safety evaluation of drug candidates. Acute toxicity is usually expressed as median lethal dose (LD_50_), which is the dose amount of a tested molecule to kill 50 % of the treated animals within a given period. According to the regulations and guidelines for the toxicity testing of pharmaceutical substances established by the Organization for Economic Co-operation and Development (OECD), the U.S. Food and Drug Administration (FDA), the National Institutes of Health (NIH), the European Agency for the Evaluation of Medicinal Products (EMEA), etc., the use of alternative in vitro or in silico toxicity assessment methods that avoid the use of animals are strongly recommended [1–4]. Moreover, in vivo testing for acute toxicity is time-consuming and costly, and therefore extensive efforts have been devoted to the development of in silico methods for toxicity.

Over past decades, a number of quantitative structure–activity relationship (QSAR) models have been developed to predict rodent acute toxicity [5–7], It is well-known that acute toxic effect results from multiple potential modes of action (MOA), and it is quite difficult to develop a universal model with reliable prediction accuracy to an extensive data set. Therefore, most QSAR models were built from small data sets of congeneric compounds [8–10] and thus had limited application domains. Recently, several theoretical models were developed based on relatively large-scale data sets with diverse compounds [9–12]. For example, Zhu et al. [10] developed five QSAR models for 7385 compounds with rat oral acute toxicity data, and the two models developed by *k*NN and RF achieved comparable performance for the test set (*r*^2^ = 0.66 and 0.70, respectively) to TOPKAT. However, in Zhu’s study, 997 molecules were identified as outliers and eliminated from the training set. Another study reported by Raevsky [13] and coworkers proposed a so-called Arithmetic Mean Toxicity (AMT) modelling approach, which produced local models based on a *k*-nearest neighbors approach. This approach gave correlation coefficients (*r*^2^) from 0.456 to 0.783 for 10,241 tested compounds, but the prediction accuracy for a molecule depended on the number and structural similarity of its neighbors with experimental data in the training set [13]. Recently, Lu et al. [14] employed local lazy learning (LLL) method to develop LD_50_ prediction models, and the rat acute toxicity of a molecule could be predicted by the experimental data of its *k* nearest neighbors. A consensus model by integrating the predictions of individual LLL models yielded a correlation coefficient *r*^2^ of 0.712 for the test set containing 2896 compounds. Similar to Raevsky’s approach [13], Lu’s approach relied on the priori knowledge of the experimental data of a query’s neighbors, and therefore, the actual prediction capability of this method was associated with the chemical diversity and structural coverage of the training set [15].

Due to the complicated mechanisms involved in acute toxicity, it is a difficult task to build a single QSAR model with reliable prediction accuracy by using traditional statistical approaches, such as multiple linear regression (MLR), partial least squares (PLS), principal components regression (PCR), etc. However, machine learning methods have shown promising potential to establish the complex QSARs for the data sets with diverse ranges of molecular structures and mechanisms. Certainly, each machine learning method has its intrinsic advantages, shortcomings, and practical constraints. Moreover, the performance of different machine learning methods depends on the structural diversity and representativeness of the molecules in the data set. Therefore, it is quite important to choose the most suitable machine learning method to develop the prediction model for a specific toxicity data set.

Among all existed machine learning methods, most of them may have the common problem of overtraining and overfitting in solving high-dimensional and complex nonlinear problems because they usually need to estimate and optimize many hyperparameters. It is well-known that the complexity of a model often grows linearly with the dimension of data, and thus some forms of post-processing are required to reduce the computational complexity. In order to solve this problem, the relevance vector machine (RVM) method introduced the Bayesian criteria into learning process, and it employs a sparse prior to reduce the unrelevant support vectors of the decision boundary in feature space and gets a sparser model accordingly. Contrary to the similar algorithm, support vector machine (SVM), the penalty parameter *C* and the insensitive-loss parameter *ε* are automatically valuated and error bars are got through covariance function in the RVM regression. Meanwhile, RVM has a comparable generalization ability, and its non-zero weights reflect prototype of sampling more than SVM. Therefore, RVM may be a good choice for QSAR modelling.

In this study, based on a large public data set containing 7385 rat oral acute toxicity data compiled by the previous study [10], RVM was employed to establish the regression models for the prediction of oral acute toxicity in rat, and was compared with the other six machine learning methods, including SVM, *k*-nearest-neighbor regression (*k*NN), random forest (RF), local approximate Gaussian process (laGP), multilayer perceptron ensemble (MPLE), and eXtreme gradient boosting (XGBoost). The performance of all the seven machine learning methods was assessed and compared by the predictive power and application domains of the models to the external test set. Moreover, the possibility to achieve better prediction of rat oral acute toxicity by combining the predictions from multiple QSAR models was explored.

## Methods

### Data set of rat oral acute toxicity

The rat oral LD_50_ data set with 7385 unique organic molecules reported by Zhu et al. [10] was used in our study. The quality of the data set, originally collected from different sources, was carefully verified. The acute toxicity of each molecule was expressed as log[1/(mol/kg)] (or pLD_50_).

The SMILES of the 7385 structures in the data set were converted into 3-D structures and optimized in Discovery Studio 2.5 molecular simulation package (DS 2.5) [16]. Here, 68 molecules were eliminated because some molecular descriptors of them could not be successfully generated by Molecular Operating Environment (MOE) 2009 molecular simulation package [17], and 3 molecules with pLD_50_ values higher than 7.0 or lower than 0, distantly distributed from the other data, were removed. The final data set contained 7314 molecules, which were randomly re-split into a training set with 4938 (67.5 %) molecules and an external test set with 2376 (32.5 %) molecules by weighing the distribution of their pLD_50_ values.

### Calculation of molecular descriptors and molecular fingerprints

Originally, 334 descriptors to characterize the physicochemical properties, molecular representations, and drug-like properties of the studied molecules were calculated by using MOE. The descriptors that had zero values or zero variance were removed. Then, the correlations across all pairs of descriptors were calculated, and the redundant descriptors with the correlation (*r*) higher than the predefined threshold (0.95) to any descriptor were removed. Finally, 230 descriptors were chosen for QSAR modeling. In addition, molecular fingerprints, which characterize the substructure features of a molecule, were used. Two sets of fingerprints, including the PubChem fingerprint (PubchemFP) with 881 substructure patterns, and substructure fingerprint (SubFP) with 307 substructure patterns, were generated by PaDEL-Descriptor software [18].

### Dimension reduction by Chi squared statistics

Dimension reduction is essential to the regression analysis of high-dimensional data. The idea in dimension reduction is to find a projection *p* to a *k*-dimensional subspace, *k* ≪ *p*, such that less information is lost. Ensemble feature selection is a subset of dimension reduction techniques that apply feature selection algorithms multiple times and combine the results into one decision. The Chi squared statistics, which is one of the most popular ensemble feature selection techniques [19, 20], was employed here to find a subset of the original descriptors. It is a non-threshold based feature selection technique and has been successfully applied in many fields [21, 22]. The Chi squared statistics compared the observed distribution of class-feature value pairs to the distribution predicted by a Chi squared random distribution, and those features distinct from this null distribution were preferred. Then, the Chi squared scores *χ*^2^ were transformed into Cramer’s *V* coefficients (Eq. 1) [23]. At last, the Cramer’s *V* coefficients were ranked and 120 or 150 features with the highest Cramer’s *V* coefficients were chosen (Eq. 2).

1 $${{\upchi }}^{2} = \mathop \sum \limits_{ij} \left( {O_{ij} - E_{ij} } \right)^{2} /E_{ij}$$

2 $$V = \sqrt {\frac{{\chi^{2} /n}}{{\hbox{min} \left( {k - 1} \right)}}}$$

where *χ*^2^ is Chi square scores, *O*_*ij*_ is the observation values from measurement, *E*_*ij*_ is the expectation values from prediction, *n* is the grand total of observations and *k* is the number of rows or columns in the contingency table.

### QSAR modeling by machine learning approaches

A variety of machine learning approaches have been used to develop regression models for the prediction of ADME and toxicities [24–28]. Here, seven machine learning methods, including *k*NN, RF, SVM, RVM, laGP, MPLE, and XGBoost, were employed for model building. Two main packages of machine learning in *R* (version 3.1.3 x64), rminer [29] and mlr [30], provide generic and object-oriented interfaces to the employed machine learning methods with good scalability. The important parameters for QSAR modeling are summarized in Table 1.

**Table 1:** Some important parameters used in QSAR modeling

Models	Hyperparameters
*k*NN	The number of predictors at each node = 1–10
RF	The number of predictors at each node = 105, the number of trees = 230
SVM (RBF)	The kernel width σ = 0.03125, the penalty parameter *C* = 2, and ε in the loss function = 0.05
RVM (Laplace)	The kernel width σ = 0.044
laGP	The initial values of lengthscale = 5, the initial values of nugget = 0.1
MPLE	The number of individual perceptrons = 18, the number of units in the hidden layer = 5–8
XGBoost	Step size shrinkage = 0.1, maximum depth of a tree = 7, the max number of iterations = 69

#### Relevance vector machine (RVM)

Relevance vector machine (RVM), pioneered by Tipping, is a sparse Bayesian learning algorithm for regression and probabilistic classification developed from the standard SVM [31, 32]. It has shown better generalization performance than SVM, but it allows avoiding the set of free parameters that SVM has. RVM acquires relevance vectors and weights (*w*) by maximizing a marginal likelihood. The products of weights and kernel functions give the structure of RVM. For a data set of input-target pairs $\{ x_{n} ,t_{n} \}_{n = 1}^{N}$, we follow the standard probabilistic formulation and assume $p(t_{n} |x)$ is Gaussian ${\mathcal{N}}(t_{n} |{\text{y}}\left( {x_{n} } \right),\sigma^{2} )$. Thus the likelihood of the training data set can be written as:

3 $$p\left( {t_{n} |w,\sigma^{2} } \right) = \left( {2\uppi\sigma^{2} } \right)^{ - N/2} { \exp }\left\{ { - \frac{1}{{2\sigma^{2} }}t -\Phi w^{2} } \right\}$$

A proper kernel function is selected to create the *N* × (*N* + 1) design matrix **Φ** with $\left[ {\phi \left( {{\mathbf{x}}_{1} } \right),\phi \left( {{\mathbf{x}}_{1} } \right), \ldots ,\phi \left( {{\mathbf{x}}_{N} } \right)} \right]^{T}$, wherein $\phi \left( {{\mathbf{x}}_{n} } \right) = \left[ {1, K\left( {{\mathbf{x}}_{n} ,{\mathbf{x}}_{1} } \right),K\left( {{\mathbf{x}}_{n} ,{\mathbf{x}}_{2} } \right), \ldots , K\left( {{\mathbf{x}}_{n} , {\mathbf{x}}_{N} } \right)} \right]^{T}$. To avoid overfitting, starting values for hyper-parameter *α* and *β* (i.e. $\sigma^{ - 2}$) are chosen to build a zero-mean Gaussian prior distribution over the weights as:

4 $$p\left( {\varvec{w} |\varvec{\alpha}} \right) = \mathop \prod \limits_{i = 0}^{N} {\mathcal{N}}(w_{i} |0,\alpha_{i}^{ - 1} )$$

Then the posterior distribution over the weights is calculated as:

5 $$p\left( {\varvec{w} |\varvec{t},\varvec{\alpha},\sigma^{2} } \right) = \left( {2{{\uppi }}} \right)^{{ - \left( {N + 1} \right)/2}} \left| {\varvec{\Sigma}} \right|^{ - 1/2} { \exp }\left\{ { - \frac{1}{2}\left( {\varvec{w} -\varvec{\mu}} \right)^{T} {\varvec{\Sigma}}^{ - 1} \left( {\varvec{w} -\varvec{\mu}} \right)} \right\}$$

where the posterior covariance and mean are shown in Eqs. (6) and (7), respectively:

6 $${\varvec{\Sigma}} = \left( {\beta {\varvec{\Phi}}^{T} {\varvec{\Phi}} + {\mathbf{A}}} \right)^{ - 1} ,$$

7 $$\varvec{\mu}= \beta {{\Sigma}} {\varvec{\Phi}}^{T} \varvec{t,}$$

with ${\mathbf{A}} = {\text{diag}}\left( {\alpha_{0} ,\alpha_{1} , \ldots ,\alpha_{N} } \right)$ and $\varvec{t} = \left( {t_{1} ,t_{2} , \ldots ,t_{N} } \right)$. The likelihood distribution over the training set can be marginalized by integrating the weights to obtain the marginal likelihood. Every hyper-parameter is iterated by type-II maximum likelihood method to maximize a posterior. In every iteration, the hyper-parameters were updated as $\alpha^{New} = {{\upgamma }}_{i} /\mu_{i}^{2}$ and $\beta^{New} = \left( {N - \Sigma_{i} \gamma_{i} } \right)/\varvec{t} - {\varvec{\Phi}}\varvec{\mu}^{2}$ ($\gamma_{i} \equiv 1 - \alpha_{i} {{\Sigma }}_{ii}$, where ${{\Sigma }}_{ii}$ is the *i*-th diagonal element of the posterior weight covariance from Eq. (6) computed with the current *α* and *β* values). Repeat application of Eqs. (6) and (7) concurrently with updating of the posterior statistics **Σ** and *μ*, until convergence criteria (*α*_*i*_ is increasing toward infinity) have been satisfied. After that we can make predictions based on the posterior distribution over the weights. In this study, a Laplacian kernel $k\left( {x_{i} ,x_{j} } \right) = { \exp }\left( { - \frac{{\left\| {x_{i} - x_{j} } \right\|}}{\sigma }} \right)$ was used as the kernel function, and the kernel width *σ* was 0.044.

#### Support vector machine (SVM) regression

Support vector machine (SVM), under the frame of Vapnik–Chervonenkis theory, [33, 34] is one of the most popular machine learning methods used in QSAR modeling [35]. Although SVM was originally developed for classification, it can also be used for regression (or function approximation). In the case of regression, the objective is to find a hyperplane with small norm while simultaneously minimize the sum of the distances from the data points to the hyperplane. In this study, the Gaussian radial basis function (RBF) was used as the kernel, and grid search was employed for the optimization of the kernel parameter *σ* [36]. The penalty parameter *C* of the error term was set to 2, and the insensitive parameter *ε* in the loss function was set to 0.05.

#### *k*-Nearest-neighbor (*k*NN) regression

*k*NN is a non-parametric learning approach for classification and regression based on the closest training examples in the feature space [37, 38]. The feature selection, the number *k* of nearest neighbors, and the shape of the distance weighting function determine the performance of a *k*NN model. Here, each molecule was eliminated from the training set and its pLD_50_ value was predicted as the inverse distance weighted average activity of the *k* most similar molecules, where the value of *k* was optimized as well (*k* = 1–10).

#### Random forest (RF)

Random forest (RF) is an ensemble learning method by combining multiple decision trees and yields the consensus predictions from individual trees [39, 40]. It randomly samples the data from the training set to construct individual trees. Each node of the tree is split using the best subset of total descriptors randomly chosen at that node. Here, a 10-puzzle heuristic searching method was used to determine the most optimal parameters in RF modelling. The number of the predictors sampled for splitting at each node was set to 105, and the number of trees to grow was set to 230.

#### Local approximate Gaussian process regression (laGP)

laGP is a parallel approximate Gaussian Process (GP) regression algorithm for big data [41, 42]. The approximation is based on finding small local designs for independent prediction at particular inputs. A Gaussian process can be used as a prior probability distribution over functions in Bayesian inference, with finite dimensional distributions defined by a mean *µ*(*x*) and positive definite covariance $K\left( {x,x^{\prime } } \right)$ for *p*-dimensional inputs *x* and $x^{\prime }$. For smoothing noisy data, a nugget (*η*/*g*) can be added to $K\left( {x,x^{\prime } } \right)$ of the isotropic process. The method involves approximating the predictive equations at the local designs *X*_*n*_(*x*) close to a particular generic location *x*, and then calculating the local maximum-likelihood estimation. Two parameters, lengthscale (*θ*) and nugget (*η*), are quite important in Gaussian process predictive modeling. The optimum values of lengthscale and nugget will be reached by looping over each *x* collecting approximate predictive equations to maximize a posterior. In this study, the initial values of lengthscale and nugget were set to 5 and 0.1, respectively.

#### Multilayer-perceptron networks ensemble (MPLE)

Multilayer-perceptron network (MPL) is an artificial feed-forward neural network model where information moves forward from the input nodes, through all hidden nodes, to the output nodes without loops. A MPLE model consists of multiple layers of neuron units, usually interconnected in a feed-forward way [43, 44]. Each neuron in one layer directly connects to the neurons of the subsequent layer, and each neuron is a perceptron with multiple layers of neuron units. To minimize the loss function, optimization is done via the Broyden–Fletcher–Goldfarb–Shanno (BFGS) algorithm. In this study, a softmax function (log-linear model) was used as the activation function. The number of individual perceptrons was 18, and the number of units in the hidden layer was 5–8.

#### eXtreme gradient boosting (XGBoost)

Gradient boosting algorithm is a machine learning technique to construct an ensemble of decision trees, and XGBoost is an efficient and scalable implementation of the gradient boosting framework [45, 46]. It develops the model in a sequential stage-wise fashion like other boosting methods do, and generalizes them by allowing optimization of an arbitrary differentiable loss function. In this study, the default parameters (step size shrinkage = 0.1, maximum depth of a tree = 7, and the max number of iterations = 69) were used.

### Evaluation and validation of the regression models

The statistical significance of each regression model was assessed by adjusted *R*^2^ ($R_{adj}^{2}$) and tenfold cross-validation *R*^2^ coefficient (*q*^2^) as shown in Eqs. (8) and (9).

8 $$\begin{aligned} R_{adj}^{2}& = 1 -(1 - R^{2} ) \times \frac{n - 1}{n - p - 1} \\ &= 1 - \left[ (SSE/( {n - p} )\right] \left[ \left(SST/( {n - 1} \right)\right] \end{aligned}$$

9 $$q^{2} = \frac{{SST{-}PRESS}}{SST}$$

where *R*^2^ is the square of the Pearson correlation coefficient, *p* is the number of the parameters in the regression equation, *SSE* is the sum of squares of errors, *SST* is the total sum of squared deviations of the dependent variable values from their means, and *PRESS* is the predictive residual sum of squares.

The conventional coefficient of determination *R*^2^ ($q_{ext}^{2}$) was used to evaluate the predictive power of each model on the external test set. The acceptability thresholds of *q*^2^ for the training set and $q_{ext}^{2}$ for the test set were both set to ≥0.5. A model is over-fitted when the difference between $R_{adj}^{2}$ and $q_{ext}^{2}$ is higher than 0.3 [47, 48].

Moreover, other two parameters, mean absolute error (MAE) and root mean square error (RMSE), were used to evaluate the quality of each model.

### Analysis of application domain (AD)

It is well known that the training set for QSAR modelling might only covers a limited fraction of the entire chemical space and the applicability of any model to the query chemicals is limited, and thus the AD for any model should be defined [49]. As a result, only a certain fraction of the chemicals in any external data set is expected to fall within the AD, and this fraction is therefore referred as the data set coverage. In this study, the Standard Deviation Distance to Model (STD-DM) approach was used to estimate the AD of each model. The detailed description of the algorithm to define AD shown in Eq. (10) has been described in previous literatures [50–53].

10 $$STD - DM\left( J \right) = STDEV\left( {y\left( J \right)} \right) = \sqrt {\frac{{\sum \left( {y\left( J \right) - \bar{y}\left( J \right)} \right)^{2} }}{N - 1}}$$

where *y*(*J*) is a quantitative value of prediction for molecule *J*, and *N* is the total number of the molecules in the test set. The margin range of AD was defined as three times of the STD-DM value [50]. When a molecule is outside the AD, the STD-DM value is high and accordingly the margin range is also high.

### Scaffold analysis of molecules with large prediction errors

The scaffolds for the 249 molecules with large prediction errors (MAE > 1.0) were examined systematically. The scaffolds for each molecule were characterized by four representations, including Murcko frameworks, ring assemblies, bridge assemblies, and the side chains attached to Murcko frameworks. Murcko frameworks developed by Bemis [54] were primarily used to characterize cyclic substructures of molecules. The definitions of these four scaffold representations have been described in previous studies [55, 56]. The scaffolds were generated by using the *Generate Fragments* component in Pipeline Pilot 7.5. The frequency of each scaffold architecture was counted, and the scaffolds were sorted by the scaffold frequency. Finally, for each scaffold with frequency equal or larger than 2, its numbers present in the training and test sets were counted.

## Results and discussions

### Property distributions of rat oral acute toxicity data

**Fig. 1:**
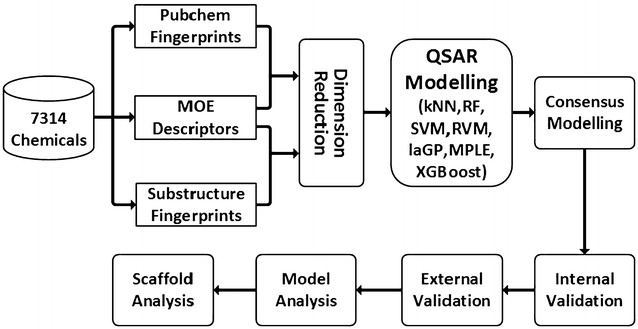
Workflow of combinatorial QSAR modelling

**Fig. 2:**
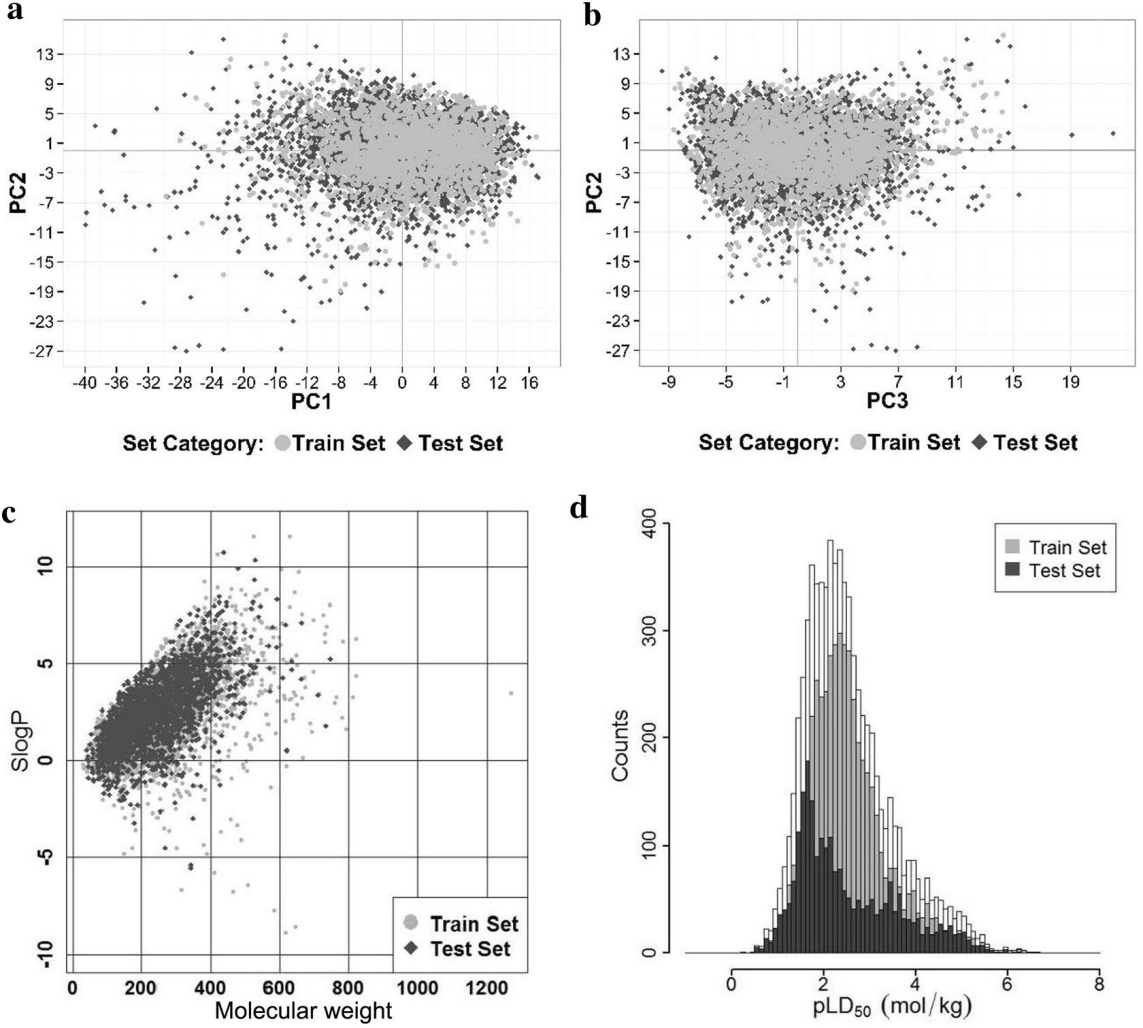
Diversity distribution of the training set (*n* = 4938) and external test set (*n* = 2376). **a**, **b** Chemical space defined by PCA factorization; **c** chemical space defined by molecular weight (MW) as X-axis and SlogP as Y-axis; **d** comparison of toxicity value distribution in different data sets. *Gray circle* stands for the training set, and black rhombus stands for the test set

In our study, 7314 organic molecules collected from the previous literature [10] were used for model development and validation. The training and test sets contained 4938 and 2376 molecules, respectively (Fig. 1). The chemical space was characterized by the scattered distributions of the first two principal components derived from the principal component analysis (PCA) for the 334 molecular descriptors and by the scattered distributions of molecule weight and Wildman and Crippen’s octanol–water partition coefficient (SlogP) [57]. As shown in Fig. 2, the chemical space of the external test set was roughly within the scope of the training set, and therefore it was feasible to predict the acute toxicity of the molecules in the test set with reasonable reliability by using the QSAR models built from the training set.

**Fig. 3:**
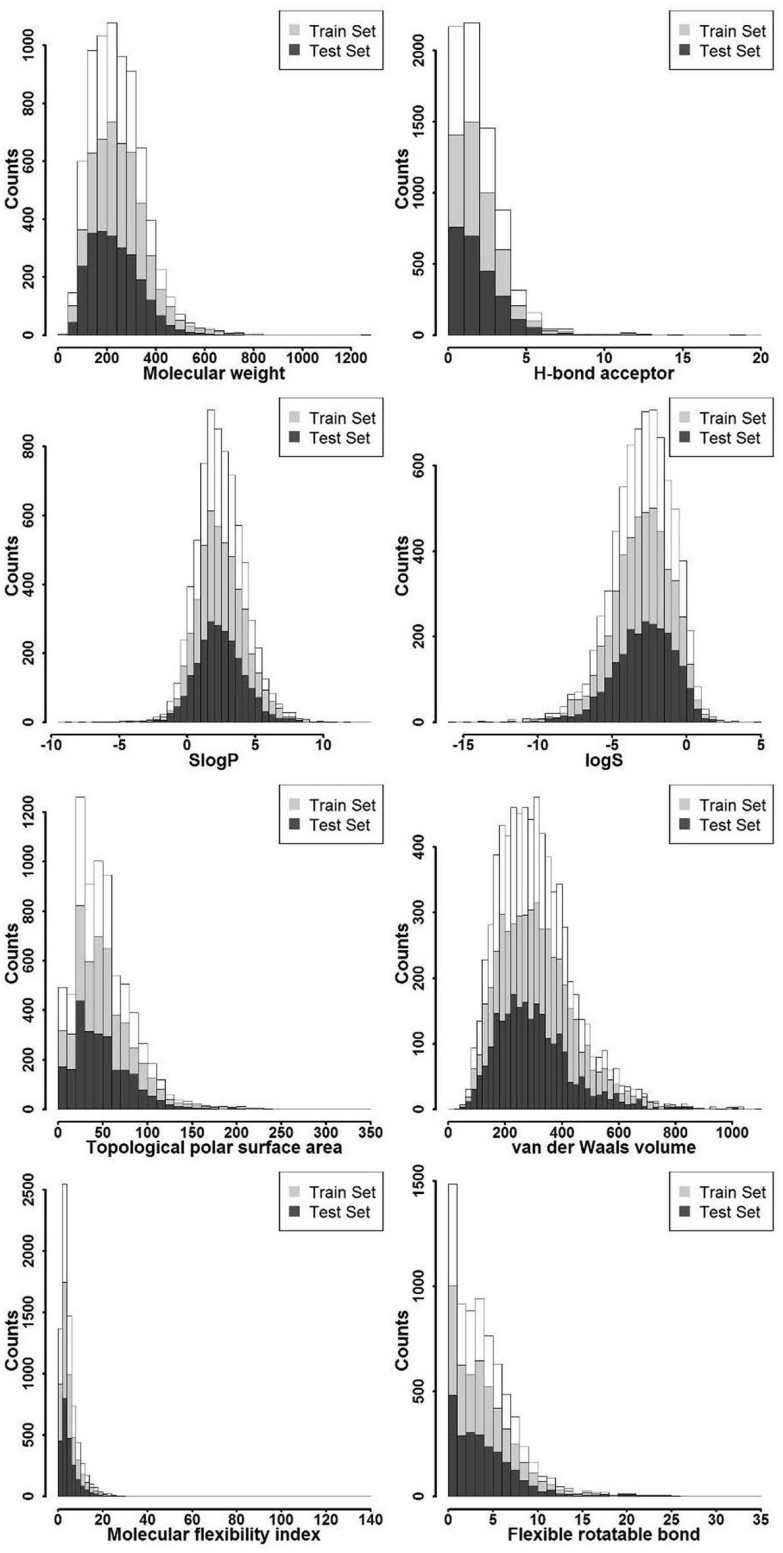
Distributions of eight studied properties

**Fig. 4:**
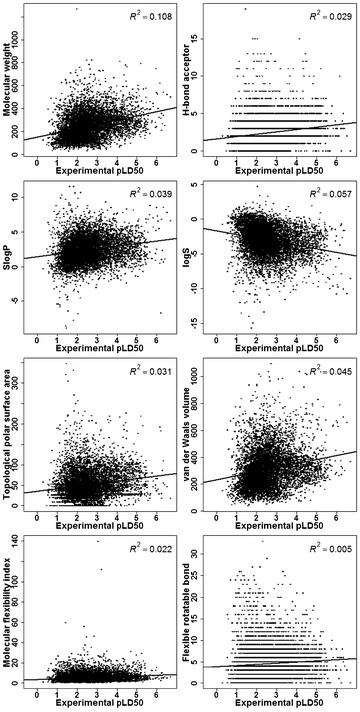
Correlations between rat oral acute toxicity and eight studied properties

The distributions of eight molecular properties for the training and test sets were shown in Fig. 3, and the correlations between rat oral acute toxicity and molecular descriptors were shown in Fig. 4. The eight molecular properties studied here, including molecular weight (MW), H-bond acceptor count (a_acc), flexible rotatable bond count (b_rotN), octanol–water partitioning coefficient (SlogP), intrinsic solubility (logS), topological polar surface area (TPSA), van der Waals volume (vdw_vol), molecular flexibility index (KierFlex), have been widely used in the prediction of ADME and toxicity [58–65].

SlogP and logS were both related to hydrophobicity. As shown in Fig. 3, the SlogP and logS values for 90 % of the compounds in the data set were less than 8 and 2, respectively. They did not show any correlation with rat oral toxicity (*R*^2^ = 0.039 and 0.057). Meanwhile, 90 % of the compounds in the database had a MW smaller than 500, and the correlation analysis showed that MW had a relatively high impact on rat oral toxicity, indicated by the slightly higher correlation (*R*^2^ = 0.108). a_acc and TPSA were usually used to represent hydrophilicity, and as shown in Fig. 4, they had worse correlations with rat oral toxicity (*R*^2^ = 0.029 and 0.031) than those related to hydrophobicity. The parameter vdw_vol accounted for the size or bulk of a molecule, and it had low correlation with rat oral toxicity (*R*^2^ = 0.045). KierFlex and b_rotN characterized the flexibility of a molecule, and both of them had no correlation with rat oral toxicity (*R*^2^ = 0.022 and 0.005). Apparently, no single descriptor showed high correlation with rat oral toxicity, and therefore rat oral toxicity could not be reliably predicted by a single or several molecular descriptors.

### Comparison of various regression models for rat oral acute toxicity

The statistical results for the training and test sets given by the QSAR models based on the MOE descriptors and two different sets of fingerprints were summarized in Tables 2, 3, 4, and 5. According to the tenfold cross validations for the training set and the predictions for the test set, the performances of the seven machine learning approaches were quite different. Apparently, among these models, the RVM models always gave the best predictions for both the training and test sets ($q_{ext}^{2} = 0.640\text{--}0.659$). The prediction capability ($q_{ext}^{2} = 0.639\text{--}0.646$) of the RF models was slightly worse than that of the RVM models but obviously better than those of the other models. The good performance of RF was not surprising because other recent studies showed that RF models generally outperformed other comparable machine learning approaches for QSAR modeling based on extensive data sets [39, 66–68]. When considering the overall statistics and prediction accuracy, RVM and RF were recommended to build the in silico models for the prediction of rat oral acute toxicity.

**Table 2:** Statistical results for the QSAR models based on 120 descriptors and Pubchem fingerprints for the test set

	*R*_adj_^2^	*q* ^2^	$q_{ext}^{2}$	RMSE_train_	MAE_train_	RMSE_test_	MAE_test_	AD coverage (%)
*k*NN	0.783	0.774	0.602	0.413	0.299	0.707	0.398	51.4
RF	0.949	0.922	0.639	0.242	0.171	0.707	0.544	81.7
SVM	0.923	0.915	0.627	0.253	0.119	0.688	0.507	58.6
RVM	0.936	0.935	0.644	0.221	0.172	0.680	0.511	62.9
laGP	0.775	0.756	0.614	0.430	0.322	0.713	0.550	72.2
MPLE	0.716	0.693	0.580	0.482	0.349	0.743	0.572	78.4
XGBoost	0.920	0.903	0.624	0.271	0.205	0.700	0.533	74.5
Consensus	0.923	NA	0.676	0.278	0.208	0.666	0.504	71.7
Consensus (Except MPLE)	0.933	NA	0.678	0.257	0.194	0.661	0.499	68.9

**Table 3:** Statistical results for the QSAR models based on 150 descriptors and Pubchem fingerprints for the test set

	*R*_adj_^2^	*q* ^2^	$q_{ext}^{2}$	RMSE_train_	MAE_train_	RMSE_test_	MAE_test_	AD coverage (%)
*k*NN	0.885	0.878	0.585	0.303	0.217	0.718	0.415	41.0
RF	0.932	0.905	0.639	0.239	0.171	0.709	0.547	82.7
SVM	0.953	0.948	0.606	0.199	0.086	0.710	0.527	67.0
RVM	0.942	0.941	0.640	0.212	0.165	0.684	0.516	64.4
laGP	0.789	0.768	0.605	0.418	0.315	0.720	0.551	73.6
MPLE	0.654	0.633	0.572	0.527	0.382	0.754	0.580	83.4
XGBoost	0.920	0.907	0.622	0.271	0.205	0.707	0.538	74.7
Consensus	0.922	NA	0.669	0.284	0.215	0.676	0.515	75.7
Consensus (Except MPLE)	0.934	NA	0.669	0.258	0.197	0.671	0.509	72.9

**Table 4:** Statistical results for the QSAR models based on 120 descriptors and Substructural fingerprints for the test set

	*R*_adj_^2^	*q* ^2^	$\varvec{q}_{{\varvec{ext}}}^{2}$	RMSE_train_	MAE_train_	RMSE_test_	MAE_test_	AD coverage (%)
*k*NN	0.815	0.805	0.636	0.383	0.277	0.674	0.364	46.1
RF	0.942	0.914	0.645	0.239	0.172	0.691	0.525	76.2
SVM	0.681	0.668	0.617	0.501	0.323	0.701	0.516	63.3
RVM	0.934	0.933	0.655	0.224	0.172	0.662	0.498	56.4
laGP	0.767	0.745	0.634	0.438	0.328	0.693	0.530	71.1
MPLE	0.679	0.656	0.596	0.509	0.374	0.729	0.558	77.0
XGBoost	0.920	0.902	0.644	0.272	0.205	0.681	0.516	67.7
Consensus	0.888	NA	0.687	0.330	0.249	0.654	0.495	69.9
Consensus (Except MPLE)	0.897	NA	0.689	0.314	0.237	0.652	0.493	68.5

**Table 5:** Statistical results for the QSAR models based on 150 descriptors and Substructural fingerprints for the test set

	*R*_adj_^2^	*q* ^2^	$q_{ext}^{2}$	RMSE_train_	MAE_train_	RMSE_test_	MAE_test_	AD coverage (%)
*k*NN	0.859	0.851	0.642	0.335	0.241	0.667	0.358	41.8
RF	0.942	0.923	0.646	0.241	0.172	0.693	0.527	77.8
SVM	0.751	0.736	0.638	0.446	0.272	0.682	0.500	58.4
RVM	0.938	0.937	0.659	0.218	0.168	0.660	0.495	55.9
laGP	0.761	0.741	0.635	0.442	0.331	0.692	0.528	68.8
MPLE	0.651	0.630	0.591	0.528	0.384	0.735	0.563	79.2
XGBoost	0.922	0.904	0.635	0.269	0.203	0.687	0.521	67.4
Consensus	0.894	NA	0.689	0.323	0.242	0.652	0.493	68.8
Consensus (Except MPLE)	0.904	NA	0.690	0.303	0.228	0.646	0.487	65.8

As shown in Tables 2, 3, 4, and 5, the MPLE models gave the lowest $q_{ext}^{2}$ (0.572–0.596) and the highest RMSE (0.729–0.754) and MAE (0.558–0.580) values for the test set, suggesting that they had the worst prediction capabilities. Meanwhile, their *R*_adj_^2^ (0.633–0.656) for the training set were always the lowest. As far as we know, our study was the first application of MPLE in QSAR modeling, and therefore we could not give our judgment to the predictive power of MPLE to different QSAR problems. However, according to our results, MPLE was not a good choice for this specific toxicity data set.

laGP is a parallelized version of the approximate Gaussian Process algorithm. Based on the molecular descriptors and PubchemFP fingerprint, the predictive power of the laGP models ($q_{ext}^{2} = 0.605\,{\text{ or}}\, \, 0.614$) was slightly better than that of the *k*NN models ($q_{ext}^{2}$ = 0.585 or 0.602) while slightly worse than that of the SVM models ($q_{ext}^{2}$ = 0.606 or 0.627). However, based on the molecular descriptors and SubFP fingerprint, the predictive power of the laGP models ($q_{ext}^{2}$ = 0.634 or 0.635) was slightly worse than that of the *k*NN models ($q_{ext}^{2}$ = 0.636 or 0.642) while slightly better than that of the SVM models ($q_{ext}^{2}$ = 0.617 or 0.638). Therefore, overall, laGP, *k*NN and SVM performed similarly to this specific toxicity endpoint.

The RVM method is quite similar to the SVM algorithm in many aspects, but it can provide a fully probabilistic output. However, up to now, little information on RVM applications in QSAR modeling has been reported in the literature. According to the data shown in Tables 2, 3, 4, and 5, we observed that the RVM models ($q_{ext}^{2}$ = 0.640 or 0.659) were obviously better than the SVM models ($q_{ext}^{2}$ = 0.606 or 0.638). Moreover, we found that the RVM modeling was more computationally efficient than the SVM modeling because RVM did not need to estimate the error/margin tradeoff parameter *C*, which might reduce the computational cost. Due to better prediction accuracy and higher computational efficiency compared with SVM, we believed that RVM should have a promising potential for the practical use in QSAR modeling in the future.

The AD coverages for the established models were summarized in Tables 2, 3, 4, and 5. The *k*NN models always showed the smallest AD coverage for the test set. Compared with the other models, the MPLE and RF models showed relatively larger AD coverages, but the RF models could give better predictions to the test set than the MPLE models. Therefore, according to the $q_{ext}^{2}$ and AD coverage, the RF models would give the best predictions for this data set.

In this study, two well-defined substructural fingerprints (SubFP and PubchemFP) were used. According to the predictions to the test set, the models based on the SubFP fingerprint (Tables 4, 5) were better than those based on the PubchemFP fingerprint (Tables 2, 3). It is possible that some fragments in SubFP were more closely related to acute toxicity than those in PubchemFP.

### Accurate prediction of rat oral acute toxicity by consensus modeling

The statistical results showed that the theoretical models using different machine learning methods have different prediction capability and model uncertainty. A useful way to reduce the model uncertainty is consensus modeling by averaging the outputs from multiple models [69–71]. Since the consensus prediction is made based on multiple different but comparable QSAR models, it may be capable of capturing the relationship between the chemical structures of the molecules and the endpoint more efficiently than a single model. Here, four consensus models were first developed by simply averaging the predictions for the test set given by the individual models shown in Tables 2, 3, 4, and 5. All the contributions of the individual models were equal, and therefore we could avoid the limitation or overemphasis of any machine learning approach. The statistical results clearly illustrated that the consensus models had higher predictive accuracy ($q_{ext}^{2}$ = 0.669–0.689) than any individual model. In addition, by comparing the MAEs given by the consensus versus individual models using the Wilcoxon test, we found that the improvement of the consensus models compared with all individual models was statistically significant (*p* < 0.01).

**Fig. 5:**
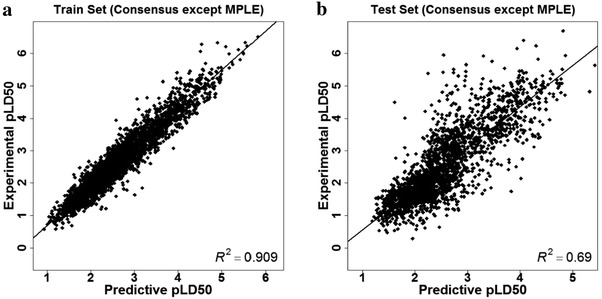
Scatter plot of the experimental pLD_50_ values versus the predicted values for the molecules in the (**a**) training and (**b**) test sets given by the consensus model without the MPLE predictions in Table 5

Because the predictions given by the MPLE models were significantly worse than those given by the other models, four consensus models were then developed without considering the predictions given by the MPLE models. As shown in Tables 2, 3, 4, and 5, all the consensus models without the MPLE predictions showed obvious performance improvement to the training set and slight performance improvement to the test set. The scatter plot of the experimental pLD50 values versus the predicted values given by the consensus model without the MPLE predictions (Table 5) for the training and test sets was shown in Fig. 5.

### Analysis of molecules with large prediction errors

As mentioned above, most prediction models had good capability for the test set, but some molecules in the test set could not be well predicted by any model or even by all models. If MAE > 1.0 was used as the criteria, the MAE of chemicals with large prediction errors given by all individual models in Table 5 ranged from 1.002 to 3.486 for the test set. In total, 575 molecules could not be well predicted by any individual model in Table 5, and 249 molecules could not be well predicted by the best consensus model in Table 5. For these 249 molecules with large prediction errors, the average experimental pLD_50_ value was 3.321, which was obviously higher than that of the molecules in the training set (2.558). Therefore, the prediction for the molecules with higher pLD_50_ values are worse than those for the molecules with lower pLD_50_ values.

The 20 molecules in the test set with the largest prediction errors by using the consensus model in Table 5 were shown in Table 6. Some of them (molecules 9, 13 and 17) had complicated structures, some of them (molecules 12, 18 and 19) have few analogues in the data set, and some of them (molecules 3, 4, 5 and 8) have phosphate groups with severe toxicity. Then, the scaffolds of the 249 molecules with large prediction errors were generated and analyzed. For the scaffolds with frequency equal or larger than 2, their numbers present in the training and test sets were counted. The scaffolds and the associated molecules in the test sets with the largest prediction errors were examined, and the representative scaffolds were summarized in Table 7. It could be observed that these scaffolds were not abundant in the data set. For example, the number of the molecules with fragment 1 in the test set was 3, and that in the training set was only 3; the number of the molecules with fragment 2 in the test set was 5, and that in the training set was only 1; the number of the molecules with fragment 10 in the test set was 3, and that in the training set was even 0. Apparently, for these scaffolds shown in Table 7, the associated molecules in the training set were quite limited and thus the established model could not give good coverage for the tested molecules with uncommon fragments.

**Table 6:** Experimental and predicted LD_50_ values for the 20 tested molecules with the largest prediction errors

No.	Structure	Exp.^a^	Fingerprints	*k*NN	RF	SVM	RVM	laGP	MPLE	XGBoost	Cons.^b^
1		5.957	PubchemFP	3.433	3.349	2.772	3.177	2.962	2.889	2.780	3.052
			SubFP	2.286	2.614	2.756	2.534	2.614	2.566	2.533	2.558
2		5.513	PubchemFP	2.590	2.529	2.461	2.694	2.514	2.743	2.638	2.596
			SubFP	2.648	2.574	2.912	2.700	2.787	2.551	2.852	2.718
3		5.658	PubchemFP	3.807	2.838	3.307	3.301	3.742	2.948	2.869	3.259
			SubFP	3.340	2.777	3.070	2.848	2.867	2.733	3.008	2.949
4		5.406	PubchemFP	1.819	2.830	2.371	2.727	2.481	2.801	3.047	2.582
			SubFP	2.976	2.609	2.839	2.637	2.736	2.755	2.662	2.745
5		5.446	PubchemFP	3.057	2.833	2.683	2.789	2.764	2.828	2.991	2.849
			SubFP	3.756	2.974	2.897	2.775	2.842	2.972	2.617	2.976
6		5.310	PubchemFP	2.588	2.595	2.589	2.861	1.843	2.817	2.564	2.551
			SubFP	2.536	2.609	3.427	3.001	2.943	2.912	2.487	2.845
7		5.307	PubchemFP	4.251	3.110	2.705	2.941	3.333	2.797	2.692	3.118
			SubFP	3.171	3.319	3.094	2.487	2.706	2.568	3.074	2.917
8		6.402	PubchemFP	3.136	3.148	2.672	2.915	3.189	3.726	2.868	3.093
			SubFP	4.263	3.692	4.778	4.332	4.084	4.073	3.292	4.073
9		6.159	PubchemFP	3.510	3.481	2.882	3.666	3.407	3.075	3.528	3.364
			SubFP	3.693	3.885	4.097	3.825	3.771	3.496	4.417	3.883
10		5.170	PubchemFP	2.641	2.758	3.202	3.004	3.119	2.816	2.821	2.909
			SubFP	2.944	2.811	3.041	2.976	2.964	2.757	2.999	2.927
11		4.019	PubchemFP	1.302	2.003	2.101	2.018	1.876	2.123	1.960	1.912
			SubFP	1.326	1.871	1.944	1.860	2.030	1.955	1.867	1.836
12		4.780	PubchemFP	2.685	2.642	2.686	2.598	2.793	2.529	2.517	2.636
			SubFP	2.666	2.548	2.980	2.663	2.640	2.454	2.540	2.642
13		4.762	PubchemFP	2.221	2.610	2.278	2.255	2.129	2.719	2.606	2.403
			SubFP	2.861	2.498	2.692	2.854	2.758	2.642	2.563	2.695
14		4.538	PubchemFP	2.284	2.576	2.483	2.533	2.575	2.921	2.165	2.505
			SubFP	2.055	2.570	2.747	2.492	2.433	2.818	2.512	2.518
15		5.006	PubchemFP	2.336	3.003	2.825	2.895	3.046	3.151	2.701	2.851
			SubFP	2.889	2.945	3.324	2.832	2.789	3.357	2.920	3.008
16		5.225	PubchemFP	3.292	3.740	3.101	3.310	3.369	3.088	3.369	3.324
			SubFP	3.580	3.144	3.396	3.200	3.322	2.991	3.153	3.255
17		1.740	PubchemFP	3.764	3.228	2.744	3.348	3.435	2.901	3.180	3.229
			SubFP	4.029	3.657	3.312	3.659	4.052	2.756	4.250	3.674
18		2.140	PubchemFP	4.421	3.509	3.397	3.555	3.886	3.466	3.482	3.674
			SubFP	4.914	3.898	3.355	3.919	4.375	3.246	3.606	3.902
19		0.291	PubchemFP	2.156	2.179	1.847	1.606	2.095	2.113	2.216	2.030
			SubFP	1.923	2.230	1.509	1.760	2.088	2.053	2.192	1.965
20		1.163	PubchemFP	3.159	2.766	2.636	2.635	3.018	2.793	1.973	2.711
			SubFP	3.743	2.757	2.547	2.829	3.033	2.669	2.047	2.804

^a^Experimental LD_50_

^b^Consensus prediction

**Table 7:** The representative scaffolds found in the tested molecules with large prediction errors (MAE > 1.0)

No.	Scaffolds	Training set			Test set			Tested molecules with large prediction errors		
		*N*	pLD_50_^a^	MAE	*N*	pLD_50_^a^	MAE	*N*	pLD_50_^a^	MAE
1		3	3.421	0.579	3	2.689	1.941	3	2.689	1.941
2		1	3.977	1.253	5	3.070	1.170	2	3.008	1.892
3		4	4.140	0.444	3	4.005	1.498	2	3.972	1.836
4		16	3.053	0.414	6	3.270	0.896	2	3.867	1.103
5		8	3.122	0.354	4	2.919	1.268	2	3.067	1.890
6		3	2.831	0.287	3	2.617	1.089	2	2.674	1.340
7		13	2.916	0.360	12	3.098	0.954	7	3.293	1.364
8		2	3.162	0.517	3	2.689	1.941	3	2.689	1.941
9		61	2.706	0.165	12	2.720	0.917	4	2.801	1.829
10		0	–	–	3	2.540	1.114	2	2.456	1.396

^a^Predicted pLD_50_ based on the best consensus model

### Analysis of important descriptors and fragments given by RVM regression models

One-dimensional sensitivity analysis was employed to evaluate the importance of the molecular descriptors and fragments for QSAR modeling, and the important descriptors were summarized in Table 8 [72]. The rings descriptor in the RF model had the highest sensitivity (0.075), and a_ICM, E_ele, vsa_pol, opr_brigid, E_nb, dipole, logS, MW, SlogP and vdw_vol were also important, indicated by relatively high sensitivity. The KierFlex descriptor in the RVM model have the highest sensitivity (0.028), and a_nF, MNDO_dipole, pmi, E_stb, E_oob, wienerPath, vsa_pol, a_acc and MW were also important, indicated by relatively high sensitivity. After examining the molecules descriptors shown in Table 8, we found that the molecular descriptors related to molecular polarity, van der Waals surface, molecular flexibility, partial charge distribution and solubility might have more contributions than the other descriptors. Furthermore, the numbers of the descriptors related to frontier molecular orbitals were relatively high, suggesting that rat oral acute toxicity might be related to molecular reactivity and intra-molecular interactions.

**Table 8:** Statistical results for the descriptors and fingerprints used in QSAR modelling

Molecular descriptors	Number of descriptors			
	120 (Descriptor + PubchemFP)	120 (Descriptor + SubFP)	150 (Descriptor + PubchemFP)	150 (Descriptor + SubFP)
2D				
Physical properties	6	7	7	7
Subdivided surface areas	8	9	10	11
Atom counts and bond counts	10	10	10	11
Kier&Hall connectivity and kappa shape indices	7	7	8	8
Adjacency and distance matrix descriptors	11	10	13	14
Pharmacophore feature descriptors	4	4	5	5
Partial charge descriptors	19	20	25	27
3D				
Potential energy descriptors	2	1	5	4
Mopac descriptors	15	15	15	15
Surface area, volume and shape descriptors	30	30	37	38
Conformation dependent charge descriptors	4	5	6	6
Fingerprints (PubchemFP)	4	–	9	–
Fingerprints (SubFP)	–	2	–	4

Moreover, after examining importance of the substructure fingerprints in the RVM models described in the Tables 3 and 5, we found that that 9 PubchemFP fragments and 4 SubFP fragments were responsible for rat oral acute toxicity. The *R*_adj_^2^ change in the stepwise regression and Cramer’s V coefficient were used to evaluate the importance of the fragments [23, 73]. The numbers of the molecules with each fragment were counted. If the number of the molecules with pLD_50_ ≥ 3 was more than that with pLD_50_ < 3, this fragment was considered to have positive contribution to high pLD_50_ and vice versa. Four PubchemFP fragments had negative contributions, and they were PubchemFP15 (the counts of nitrogen atoms ≥2), PubchemFP442 (*N*-ethylimino group), PubchemFP418 (carbon–nitrogen double bond) and PubchemFP14 (the counts of nitrogen atoms ≥1). Meanwhile, five PubchemFP fragments gave positive contributions, and they were PubchemFP400 (aromatic C–NH–C bond), PubchemFP359 (aromatic 1,3-diazacyclo group with 2-carbonous substituent), PubchemFP770 (ortho aryl nitrogen) PubchemFP833 (ortho alicyclic nitrogen) and PubchemFP527 (aromatic C–C–NH bond). On the other hand, all the four SubFP fragments had positive contributions, and they were trifluoromethyl, alkylfluoride, hetero N basic H and heterocyclic. The structures of these representative fragments were summarized in Tables 9 and 10.

**Table 9:** Nine PubchemFP fragment alerts and representative structures

No.	Fingerprint	Fragment	Description	Bit substructure	*R*_adj_^2^ change	Cramer’s V	Representative structure
*Positive fragment alerts*							
1	PubchemFP400		Detailed atom neighborhoods	N(~ H)(:C)(:C)	0.00086	0.15810	
2	PubchemFP359		Simple atom nearest neighbors	C(~ C)(:N)(:N)	0.00162	0.15641	
4	PubchemFP770		Complex SMARTS patterns	Nc1c(N)cccc1	0.00167	0.14722	
5	PubchemFP833		Complex SMARTS patterns	NC1C(N)CCCC1	0.00100	0.14504	
6	PubchemFP527		Simple SMARTS patterns	C:C:N-[#1]	0.00036	0.13989	
*Negative fragment alerts*							
1	PubchemFP15	Counts of N ≥ 2	Hierarchic element counts	≥2 N	0.00174	0.16108	
2	PubchemFP442		Detailed atom neighborhoods	C(–C)(=N)	0.00024	0.15474	
3	PubchemFP418		Simple SMARTS patterns	C=N	0.00094	0.13903	
	PubchemFP14	Counts of N ≥ 1	Hierarchic element counts	≥1 N	0.00002	0.13650

**Table 10:** Four SubFP fragment alerts and representative structures

No.	Fingerprint	Fragment	Description	SMILES	*R*_adj_^2^ change	Cramer’s V	Representative structure
1	SubFP294		Trifluoromethyl	[FX1][CX4;!$([H0][Cl,Br,I]);!$([F][C]([F])([F])[F])]([FX1])([FX1])	0.00173	0.15737	
2	SubFP9		Alkylfluoride	[FX1][CX4]	0.00024	0.15386	
3	SubFP179		Hetero N basic H	[nX3H1 + 0]	0.00161	0.14669	
4	SubFP275		Heterocyclic	[!#6;!R0]	0.00002	0.14306	

The PubchemFP fragments found in the models are relatively small, but they might be important components for toxicophores that were not defined in the fingerprint dictionary. In the SubFP fragment alerts, trifluoromethyl and alkylfluoride were often constituent parts of toxic substances, but hetero N and heterocycle might be only the background noise of models, or they may be parts of some toxic substructures not defined in the fingerprint dictionary. As been mentioned in the previous literature [14, 74, 75], some toxic chemicals contained trifluoromethyl and alkylfluoride fragments such as 2-(trifluoromethyl)-benzimidazole, which were not defined in the fingerprint dictionary and were substructures of many antitumor drugs, antibiotics, antiparasitics and ionic liquids [76–80]. In addition, some important substructures in toxicophores, such as organophosphates, organochlorines and norbornene derivates, did not exist in the PubchemFP dictionary. The phosphonic groups could be found in the SubFP dictionary, but they were only found in limited molecules and therefore disappeared through dimension reduction. Our calculations suggested that more specific and diverse fingerprints were essential and important for toxicity QSAR modeling.

## Conclusions

In this study, on the basis of a comprehensive data set of rat oral acute toxicity, the relationships between eight important molecular properties and acute toxicity were examined. We observed that rat oral toxicity could not be reliably predicted by a single or several molecular properties. Then, seven machine learning approaches were used to establish the QSAR models for oral acute toxicity. Considering the overall prediction accuracy for the test set, the RF and RVM methods outperformed the others. The consensus model by integrating the outputs from multiple individual models demonstrated better predictivity ($q_{ext}^{2}$ = 0.669–0.689) than any individual model for the test set. Our study also demonstrated that QSAR modeling based on structure fingerprints could afford potential important substructural fragments as toxicity alerts, but a proper and enough large fingerprint dictionary should be adopted. By scaffold analysis, we found that quite limited numbers of molecules with certain scaffolds in the training set would reduce the prediction accuracy of the models. According to the results of this study, we believed that the successful modeling methods used here could be employed for other toxicity endpoints.
